# Identification of Integrated Stress Response–Related Biomarkers in Sepsis‐Induced Acute Respiratory Distress Syndrome

**DOI:** 10.1155/mi/3190064

**Published:** 2026-09-07

**Authors:** Ye Gao, Ni Wang, Zhaoyang Xiao

**Affiliations:** ^1^ Department of Anesthesiology, The Second Affiliated Hospital of Dalian Medical University, Dalian, Liaoning, 116000, China, dlmedu.edu.cn

**Keywords:** acute respiratory distress syndrome (ARDS), integrated stress response, machine learning, sepsis, single-cell RNA sequencing

## Abstract

**Background:**

Sepsis‐induced acute respiratory distress syndrome (ARDS) is a fatal inflammatory lung injury. The integrated stress response (ISR) may play stage‐dependent roles in lung injury, but its clinical relevance in sepsis‐induced ARDS remains unclear. We aimed to identify ISR‐related biomarkers and explore their potential mechanisms.

**Methods:**

Public transcriptomic datasets of sepsis‐induced ARDS were analyzed. Peripheral blood mononuclear cell (PBMC) single‐cell RNA sequencing (scRNA‐seq) was used to define key immune cell populations and intercellular communication. Differential expression analyses combined with machine learning were applied to screen ISR‐associated biomarkers, and a nomogram was developed as an exploratory tool for ARDS risk stratification. Biomarker expression was validated in a cecal ligation and puncture (CLP)‐based rat model representing early sepsis and ARDS‐like lung injury. Lung injury was assessed by light and electron microscopy, histological scoring, lung wet/dry weight ratio, and BALF total protein measurement. Gene expression was quantified by qPCR and protein levels by Western blotting. Functional enrichment, immune infiltration, and trajectory analyses were performed to investigate underlying pathways and immune dynamics. The biomarkers were further validated in an independent external dataset.

**Results:**

Nuclear factor erythroid 2‐like 2 (NFE2L2) and ZFP36L1 were identified as potential ISR‐associated biomarkers in sepsis‐induced ARDS, and the two‐gene nomogram showed good predictive performance. In vivo, peripheral blood Tnf and Il6 mRNA expression increased after CLP, while rats with ARDS‐like lung injury exhibited marked lung injury and reduced tight junction markers. Nfe2l2 and Zfp36l1 mRNA levels were elevated in early sepsis but decreased in established ARDS‐like lung injury, while their protein levels were generally reduced in established ARDS‐like lung injury compared with early sepsis. Canonical ISR activation markers, including the p‐eukaryotic translation initiation factor 2α (eIF2α)/total eIF2α ratio and Atf4, Ddit3/Chop, and Ppp1r15 a/Gadd34 expression, progressively increased from early sepsis to ARDS‐like lung injury. Enrichment analyses implicated multiple ISR‐related pathways. Immune infiltration analysis revealed altered immune cell composition, with NFE2L2 negatively correlated with activated B cell infiltration. Single‐cell trajectory analysis demonstrated dynamic expression of these biomarkers during differentiation of key immune cell subsets.

**Conclusion:**

NFE2L2 and ZFP36L1 are potential ISR‐associated biomarkers in sepsis‐induced ARDS and may contribute to disease progression through stress‐response regulation and immune modulation, supporting biomarker‐driven risk stratification and providing a foundation for future mechanistic and interventional studies.

## 1. Introduction

Acute lung injury (ALI) and acute respiratory distress syndrome (ARDS) caused by sepsis are common and critical conditions characterized by severe respiratory distress and refractory hypoxemia [1]. ARDS, a severe manifestation of ALI, is one of the leading causes of respiratory failure and multiple organ dysfunction, posing a significant threat to the survival of critically ill patients [2], with reported mortality rates ranging from 50% to 68.8% [3, 4]. Despite substantial advances in critical care medicine, outcomes for ALI/ARDS remain unsatisfactory [5]. Current treatment strategies, such as management of the underlying disease, respiratory support, pharmacotherapy, and fluid management, often yield limited therapeutic benefit [6–9]. This limited effectiveness is largely attributed to the complex and multifactorial pathogenesis of ALI/ARDS [10, 11]. Therefore, identifying biologically relevant biomarkers holds great clinical significance for improving the diagnosis and treatment of ARDS.

The integrated stress response (ISR) is an adaptive, self‐protective mechanism by which cells respond to internal and external stressors. By finely regulating the translational machinery, ISR prioritizes the synthesis of critical proteins while suppressing the production of nonessential proteins, thereby sustaining essential cellular functions. Canonical ISR activation is mainly mediated by phosphorylation of eukaryotic translation initiation factor 2α (eIF2α) through stress‐responsive kinases, such as PERK, GCN2, PKR, and HRI, which are activated in response to diverse stressors such as metabolic dysregulation, amino acid imbalance, viral infection, heme deficiency, oxidative stress, and mitochondrial or endoplasmic reticulum stress [12, 13]. Notably, the cascade of pathological changes induced by ARDS is closely related to the ISR [14]. In the early phase of ARDS, ISR activation may represent an adaptive protective response that helps injured epithelial, endothelial, and immune cells restore proteostasis and limit stress‐related damage [12, 13]. However, persistent or excessive ISR activation may become maladaptive by promoting sustained inflammation, epithelial and endothelial barrier dysfunction, cell death, and impaired tissue repair[14–16]. In ARDS, inflammation mediated by mononuclear macrophages and CD4^+^ T cells not only directly contributes to lung tissue injury but also serves as a critical trigger for the activation of ISR pathways [15, 16]. Therefore, dynamic ISR activation may be closely related to ARDS progression, risk stratification, and therapeutic responsiveness, yet robust biomarkers connecting ISR dynamics with sepsis‐induced ARDS remain insufficiently defined.

In this study, we integrated single‐cell and transcriptome datasets with ISR‐related gene (ISR‐RG) signatures associated with both sepsis‐induced ARDS and ISR. Using single‐cell analysis, differential expression profiling, machine learning, and expression validation, we systematically screened and verified candidate biomarkers. Furthermore, we validated these biomarkers in a cecal ligation and puncture (CLP)‐based rat model representing sepsis‐induced ARDS‐like lung injury and explored their mechanistic roles at the single‐cell level. Collectively, our findings provide novel insights that may inform future diagnostic and therapeutic strategies for ARDS.

## 2. Materials and Methods

### 2.1. Data Acquisition

The GEOquery package (v2.74.0) [17] was employed to retrieve datasets related to ARDS from the Gene Expression Omnibus (GEO) database (https://www.ncbi.nlm.nih.gov/geo/). Specifically, the training set GSE32707 comprised blood samples from 58 septic patients (control group) and 31 patients with sepsis‐induced ARDS (ARDS group). This dataset is based on microarray data generated using the GPL10558 platform. The validation dataset GSE66890 included 28 blood samples from septic patients and 29 from patients with sepsis‐induced ARDS, with microarray data generated on the GPL6244 platform. In addition, the single‐cell RNA sequencing (scRNA‐seq) dataset GSE151263 (platform: GPL20301) contained peripheral blood mononuclear cell (PBMC) samples from four septic patients and three patients with sepsis‐induced ARDS. Detailed timing information for blood sampling relative to sepsis diagnosis, ARDS onset, ICU admission, or initiation of mechanical ventilation was not uniformly available in the public datasets. Therefore, these clinical transcriptomic datasets were analyzed as cross‐sectional datasets rather than longitudinal cohorts. Finally, based on previously published literature [18], a total of 529 ISR‐RGs were compiled after removing duplicates (Table S1).

### 2.2. scRNA‐Seq Analysis

For the GSE151263 dataset, the Seurat package (v5.0.1) [19] was used for data processing and quality control (unless otherwise specified, all tools referenced in this subsection refer to functions in this package). Quality control criteria included retaining cells expressing more than 200 genes, genes detected in at least three cells, and filtering out cells with fewer than 200 or more than 2000 detected genes (nFeature_RNA) or total counts below 500 or above 10,000 (nCount_RNA). Cells with a mitochondrial gene content exceeding 10% (percent_mt) were excluded. Normalization was performed using the NormalizeData function in Seurat. Highly variable genes were identified using FindVariableFeatures, with the top 2000 selected for downstream analyses and visualized using LabelPoints.

Principal component analysis (PCA) was performed using RunPCA, and significant principal components (PCs) were identified using the JackStraw function (*p* < 0.05). Unsupervised clustering was conducted with FindNeighbors and FindClusters, and dimensionality reduction for visualization was achieved using RunUMAP on the selected PCs (resolution = 0.5). Marker genes for each cell cluster were identified using FindAllMarkers (|log_2_ fold change (FC)| > 0.25, adj.*p*  < 0.05), and annotated based on the CellMarker database (http://117.50.127.228/CellMarker/) combined with relevant literature [20]. Expression patterns of marker genes for different cell types were visualized, and the distribution of cell types in ARDS and control samples was depicted using the ggplot2 package (v3.3.6) [21]. Key cell types were selected for further analysis based on literature reports of their relevance to ARDS pathogenesis and biological interest. To explore the biological functions of these key cell types, pathway enrichment analysis was performed using the ReactomeGSA package (v1.12.0) [22]. Enrichment results were extracted with the pathways function, and the top 10 significant pathways were visualized. To further investigate biomarker‐related mechanisms in key cell types, the FeaturePlot function in the Seurat package (v5.0.1) was employed to visualize the distribution of candidate biomarkers across cell types. The Wilcoxon test was used to compare biomarker expression levels between ARDS and control samples within each cell type. Intercellular communication was analyzed using the CellChat package (v1.6.1) [23], which infers ligand‐receptor interactions to reveal signaling pathways mediating communication among cell populations.

### 2.3. Differential Expression and Enrichment Analyses

To identify differentially expressed genes (DEGs) in the key cell types (DEGs‐KC), the FindMarkers function in the Seurat package (v5.0.1) was used to perform differential expression analysis on key cell subsets from GSE151263 (|avg_log_2_FC| > 0.1, min. pct = 0.25, *p*  < 0.05). For the bulk expression dataset GSE32707, the limma package (v3.54.0) [24] was employed to identify DEGs between ARDS and control samples (|log_2_FC| ≥ 0.25, *p*  < 0.05). The ggplot2 (v3.3.6) and pheatmap (v1.0.12) [25] packages were used to generate a volcano plot and a heatmap, respectively. The top 10 upregulated and top 10 downregulated genes (by |log_2_FC|) were highlighted in the volcano plot, and their expression patterns were visualized in the heatmap. To identify potential ISR‐related biomarkers, the ggvenn package (v0.1.9) [26] was used to intersect ISR‐RGs, DEGs‐KC, and bulk DEGs, resulting in a set of candidate genes. To gain insight into the biological pathways associated with these candidates, the clusterProfiler package (v4.6.2) [27] was employed to perform Gene Ontology (GO) and Kyoto Encyclopedia of Genes and Genomes (KEGG) enrichment analyses (*p* < 0.05). The top five significant GO terms for each category (biological process [BP], cellular component [CC], and molecular function [MF]) and all significant KEGG pathways were visualized.

### 2.4. Machine Learning and Expression Validation

To further refine the candidate genes and identify robust feature genes, the glmnet package (v4.1.4) [28] was used to perform 10‐fold cross‐validation least absolute shrinkage and selection operator (LASSO) regression analysis. Genes with non‐zero coefficients in the optimal model (lowest mean squared error) were retained as LASSO‐derived feature genes. Meanwhile, the support vector machine recursive feature elimination (SVM‐RFE) algorithm in the caret package (v6.0.93) [29] was employed to select additional feature genes based on the SVM‐RFE method with five‐fold cross‐validation, retaining genes at the point of maximum model accuracy. The ggvenn package (v0.1.9) was used to intersect the LASSO and SVM‐RFE results, and overlapping genes were considered as candidate biomarkers. Next, the expression levels of these candidate biomarkers were validated using the Wilcoxon test in both GSE32707 and GSE66890 datasets (*p*  < 0.05). Genes with significant differential expression and consistent expression trends between ARDS and control samples in both datasets were retained as final biomarkers.

### 2.5. Construction and Evaluation of the Nomogram

To evaluate the predictive utility of the identified biomarkers for ARDS, a nomogram was constructed based on the GSE32707 dataset using the rms package (v6.5.0) [30]. Model calibration was assessed using the calibrate package (v1.7.7) (https://www.rdocumentation.org/packages/calibrate/versions/1.7.7), and the diagnostic performance and clinical applicability were further evaluated by plotting a clinical impact curve (CIC) using the rmda package (v1.6) [31]. The nomogram was designed as an exploratory model for risk stratification rather than a clinical decision‐making tool.

### 2.6. Gene Set Variation Analysis (GSVA) and Gene Set Enrichment Analysis (GSEA)

To explore the functional pathways involved in the progression of ARDS, GSVA was conducted. The background gene set “c2.all.v2023.2.Hs.symbols” was obtained from the Molecular Signatures Database (MSigDB, https://www.gsea-msigdb.org/gsea/msigdb). The GSVA package (v1.46.0) [32] was used to calculate enrichment scores for signaling pathways in all samples from GSE32707. The Wilcoxon test was then applied to assess the significant differences in pathway enrichment scores between the ARDS and control groups. Pathways with *p*‐values <0.05 were considered significantly different and were ranked in descending order by |*t* score|. All significant pathways were visualized.

Moreover, to further investigate the potential functions of the identified biomarkers, GSEA was conducted. Specifically, for GSE32707, the psych package (v2.2.9) [33] was used to determine the correlations between each biomarker and other genes. Genes were ranked from the highest to lowest correlation to each biomarker. The background gene set “c2.cp.kegg.v2023.1.Hs.symbols” was downloaded from MSigDB, and clusterProfiler package (v4.6.2) was used to perform GSEA (|normalized enrichment score (NES)| > 1, *p*  < 0.05).

### 2.7. Immune Infiltration Analysis

To understand alterations in the immune microenvironment in ARDS patients, an immune infiltration analysis was conducted. Based on 28 immune cell types [34], the ssGSEA algorithm in the GSVA package (v1.46.0) was used to calculate enrichment scores for each immune cell type in GSE32707. The results were visualized using the pheatmap package (v1.0.12). The Wilcoxon test was applied to identify immune cell types with significantly different abundances between ARDS and control samples (*p* < 0.05). Furthermore, the psych package (v2.2.9) was employed to analyze the correlations among differential immune cell types, as well as between differential immune cells and the identified biomarkers (|correlation coefficients| > 0.3, *p*  < 0.05).

### 2.8. Gene–Gene Interaction (GGI) Network, Chromosome, and Subcellular Localization Analyses

To predict genes functionally associated with the identified biomarkers, GeneMANIA (http://genemania.org/) was used to construct GGI networks. The RCircos package (v1.2.2) [35] was employed to map the chromosomal locations of the biomarkers, providing a basis for subsequent genomic studies. Finally, since proteins must be localized to specific subcellular compartments to exert their biological functions, the subcellular localization of each biomarker was determined using the UniProt database (https://www.uniprot.org/). The resulting localization information was visualized using Cytoscape (v3.10.0) [36].

### 2.9. Construction of the Regulatory Network

To investigate the potential regulatory mechanisms governing the expression of the identified biomarkers, the miRDB (https://mirdb.org/), miRanda (http://mirtoolsgallery.tech/), and TargetScan (https://www.targetscan.org) databases were queried using the multiMiR package (v1.20.0) [37] to predict microRNAs (miRNAs) targeting the biomarkers. The intersection of the predicted miRNAs from all three databases was then taken to identify common target miRNAs. Based on these, the miRNet database (https://www.mirnet.ca/) was employed to predict long noncoding RNAs (lncRNAs) interacting with the target miRNAs.

Moreover, to identify transcription factors (TFs) that may regulate the biomarkers, the NetworkAnalyst database (https://www.networkanalyst.ca/NetworkAnalyst/) was used to predict TF‐biomarker interactions. All regulatory relationships were visualized using Cytoscape (v3.10.0).

### 2.10. Pseudotime Analysis

To explore the dynamic expression patterns of biomarkers during key cell differentiation, pseudotime trajectory analysis was performed. The RunUMAP function was first used to re‐dimensionalize key cell types. Subsequently, the Monocle package (v2.26.0) [38] was applied to reconstruct the cell differentiation trajectories and simulate the developmental progression of key cells.

### 2.11. Animals and Treatment

Healthy male Sprague–Dawley (SD) rats (220–250 g, 8–10 weeks old) were obtained from the Laboratory Animal Center of Dalian Medical University. Rats were housed under controlled temperature and humidity with free access to food and water. All procedures were approved by the Laboratory Animal Ethics Committee of Dalian Medical University (Approval No. XL250911369) and conducted in accordance with the NIH Guide for the Care and Use of Laboratory Animals. For all surgical procedures, rats were anesthetized with sevoflurane delivered in oxygen using an induction concentration of 5% and a maintenance concentration of 2%–3%. The depth of anesthesia was monitored by the pedal withdrawal reflex, respiratory rate, and mucosal color throughout the procedure. At the end of each experiment, rats were euthanized by maintaining sevoflurane at >5% until the loss of all reflexes, followed by cervical dislocation, in accordance with the 2020 AVMA Guidelines for the Euthanasia of Animals. Rats were randomly assigned into three groups (*n* = 4 per group; subsets of samples were used for specific molecular assays as indicated in the figure legends): (1) control group (C): sham surgery without CLP; (2) CLP group (P): subjected to CLP and sacrificed at 8 h; and (3) CLP + ARDS‐like lung injury group (S): subjected to CLP and monitored for the development of ARDS‐like lung injury, followed by sacrifice at 18 h.

The CLP procedure was performed as a moderate‐to‐severe sepsis model. Briefly, after anesthesia, the abdomen was shaved and disinfected, and a midline laparotomy was performed. The cecum was carefully exteriorized, and approximately 50% of the cecum distal to the ileocecal valve was ligated with a sterile silk suture, avoiding intestinal obstruction. The ligated cecum was punctured once with an 18‐gauge needle through‐and‐through, and a small amount of fecal material was gently extruded to ensure patency of the puncture site. The cecum was then returned to the abdominal cavity, and the abdominal wall and skin were closed in layers. After surgery, rats received subcutaneous fluid resuscitation with sterile saline at 50 mL/kg. Lidocaine cream was topically applied to the surgical wound for postoperative analgesia. No antibiotics were administered. Sham‐operated rats underwent the same anesthesia, laparotomy, and cecal exteriorization procedures but without cecal ligation or puncture. No animals died before the scheduled sampling time points. In this model, the 8‐h time point was used to represent early CLP‐induced sepsis with systemic inflammatory activation, whereas the 18‐h time point was used to represent established CLP‐induced ARDS‐like lung injury. Systemic inflammatory activation was supported by increased peripheral blood Tnf and Il6 mRNA expression, whereas ARDS‐like lung injury was supported by histological and ultrastructural lung injury, increased lung injury score, elevated lung wet/dry weight ratio, increased BALF total protein concentration, and decreased tight junction marker expression in lung tissues.

### 2.12. Optical Microscopy and Transmission Electron Microscopy Observation

After euthanasia, lung tissue samples were collected, fixed, embedded, sectioned, and stained with hematoxylin and eosin. Histological changes were examined under a light microscope. Lung injury was semi‐quantitatively scored based on four pathological features: alveolar septal thickening, inflammatory cell infiltration, alveolar and interstitial edema, and alveolar hemorrhage or structural destruction. Each parameter was scored from 0 to 4 according to injury severity, and the total score for each field ranged from 0 to 16. For each animal, five nonoverlapping fields were randomly selected at 400× magnification, and the mean score was calculated. Scoring was independently performed by two investigators blinded to the experimental groups, and the average of the two scores was used as the final lung injury score for each animal.

For transmission electron microscopy, lung tissue tips were excised and cut into 1 mm^3^ blocks. Five tissue blocks per rat were randomly selected for observation. Samples were examined under an EM‐1200EX transmission electron microscope at 20,000× magnification. For each block, five random fields of view were assessed to observe lesions in vascular endothelial cells and alveolar epithelial cells.

### 2.13. Lung Wet/Dry Weight Ratio

The lung wet/dry weight ratio was measured to assess pulmonary edema. After euthanasia, a portion of the right lung was collected immediately and gently blotted to remove surface blood and excess fluid. The tissue was weighed to obtain the wet weight and then dried in an oven at 60°C for 72 h until a constant weight was achieved. The dried tissue was weighed again to obtain the dry weight. The lung wet/dry weight ratio was calculated as the wet weight divided by the dry weight. Four animals per group were used for this analysis.

### 2.14. BALF Collection and Total Protein Measurement

BALF total protein concentration was assessed to evaluate the disruption of the alveolar‐capillary barrier. Following euthanasia, the trachea was carefully exposed and intubated, and bronchoalveolar lavage was carried out before lung harvest. The lungs were flushed three times with 1 mL of freshly prepared cold sterile PBS. The lavage returns were combined and centrifuged at 4°C to pellet the cells and debris. The resulting supernatant was collected for protein quantification using a BCA protein assay kit (Beyotime Biotechnology, P0012, Shanghai, China) following the manufacturer’s protocol. Measurements were performed in duplicate for each sample, and the mean of the duplicate readings was used as the biological replicate. Four animals were analyzed in each group.

### 2.15. Quantitative Real‐Time PCR Analysis

Total RNA was isolated from peripheral blood samples for Tnf and Il6 analysis and from lung tissue samples for Tjp1, Cldn5, nuclear factor erythroid 2‐like 2 (Nfe2l2), Zfp36l1, Atf4, Ddit3, and Ppp1r15a analysis using the TRIzol reagent (GLPBIO) according to the manufacturer’s instructions, and RNA concentration was determined using a NanoDrop 2000 spectrophotometer (Thermo Scientific). First‐strand cDNA was synthesized from 1 μg of total RNA using the HiScript 1^st^ Strand cDNA Synthesis Kit (Vazyme Biotech, Nanjing, China). The expression levels of Tnf and Il6 in peripheral blood samples and Tjp1, Cldn5, Nfe2l2, Zfp36l1, Atf4, Ddit3, and Ppp1r15a in lung tissue samples were quantified by real‐time PCR using the HiScript II One Step SYBR Green Kit (Vazyme Biotech) on a StepOne real‐time PCR system. Relative gene expression was normalized to the endogenous control GAPDH using the comparative Ct (ΔΔCt) method. Primer sequences were as follows:

Tnf: 5ʹ‐CCAACAAGGAGGAGAAGT‐3ʹ (forward primer), 5ʹ‐GTATGAAATGGCAAATCG‐3ʹ (reverse primer); Il6: 5′‐AGTTGTGCAATGGCAATTCTGA‐3′ (forward primer), 5′‐CTCTGGCTTTGTCTTTCTTGTTATCTTT‐3′ (reverse primer); Tjp1: 5′‐GATGAGCGGGCTACCTTA‐3′ (forward primer), 5′‐ATGGGAGCGAACTGAATG‐3′ (reverse primer); Cldn5: 5′‐TTGGAAGGGGCTGTGGATG‐3′ (forward primer), 5′‐TGAGCGCCGGTCAAGGTAA‐3′ (reverse primer); Nfe2l2:

5′‐GTTGCCGCTCAGAACTGT‐3′ (forward primer), 5′‐TTCCCATCCTCATCACGT‐3′ (reverse primer); Zfp36l1: 5′‐GGGGACAAGTGCCAGTTCG‐3′ (forward primer), 5′‐GCTCCTCGGCGTTGTGAAT‐3′ (reverse primer); Atf4: 5′‐AGGTGTTCCTCGTGGTTC‐3′ (forward primer), 5′‐CCTAGTAGCTGCTGTCTTGTT‐3′ (reverse primer); Ddit3: 5′‐CACAAGCACCTCCCAAAG‐3′ (forward primer), 5′‐CCTGCTCCTTCTCCTTCAT‐3′ (reverse primer); Ppp1r15a: 5′‐AGACAATAAGGCTGAACCC‐3′ (forward primer), 5′‐CTGCCAGAACAGACAGAAGT‐3′ (reverse primer); and GAPDH: 5′‐TGCCTCGCTTCACCACCTTCT‐3′ (forward primer), 5′‐AGGCCGGTGCTGAGTATGTC‐3′ (reverse primer).

### 2.16. Western Blot Analysis

Total protein was extracted from the lung tissue using whole cell lysis buffer. Protein samples were separated by sodium dodecyl sulfate–polyacrylamide gel electrophoresis (SDS–PAGE) and transferred onto polyvinylidene fluoride (PVDF) membranes by wet transfer. After blocking, membranes were incubated with anti‐NFE2L2/NRF2 antibody (Absin, Cat. No. abs120634; 1:2000), anti‐ZFP36L1 antibody (Absin, Cat. No. abs145715; 1:2000), anti‐phospho‐eIF2α (Ser51; Cell Signaling Technology, Cat. No. 3398; 1:1000), anti‐total eIF2α (Cell Signaling Technology, Cat. No. 5324; 1:1000), and anti‐β‐actin antibody (Abcam, Cat. No. ab8227; 1:5000). For ISR activation analysis, phospho‐eIF2α and total eIF2α were detected on parallel membranes loaded with the same protein samples, with β‐actin used as the loading control for each membrane. Detection was performed using horseradish peroxidase‐conjugated goat anti‐rabbit IgG secondary antibody (Abcam, Cat. No. ab205718; 1:5000). Band intensities were quantified using ImageJ software. NFE2L2 and ZFP36L1 protein levels were normalized to β‐actin. The p‐eIF2α/total eIF2α ratio was calculated after the normalization of phospho‐eIF2α and total eIF2α signals to their corresponding β‐actin controls.

### 2.17. Statistical Analysis

Bioinformatics analyses were conducted using R software (v4.2.2). First, differential expression analysis was conducted with limma (v3.54.0) [24], and functional enrichment and GSEA analyses were completed using clusterProfiler (v4.6.2) and fgsea (v1.24.0). Next, in the machine learning phase, LASSO regression and SVM‐RFE feature selection were carried out using glmnet (v4.1‐4) [28] and caret (v6.0‐93). The clinical model was evaluated using nomogram analysis with the rms package (v6.5‐0) and decision curve analysis with the rmda package (v1.6) [31]. In the single‐cell‐level analysis, harmony (v1.0) was applied for single‐cell data integration, Seurat (v5.0.1) was used for cell annotation, and CellChat (v1.6.1) [23] and monocle (v2.26.0) were employed to analyze intercellular communication and pseudotime trajectories. The Wilcoxon test was used for nonparametric comparisons where appropriate. These analyses were performed using established bioinformatics and statistical methods [39–41].

Statistical analyses of experimental data were performed using SPSS 14.0, and figures were generated with GraphPad Prism 5. Differences among the three experimental groups were assessed by one‐way analysis of variance (ANOVA), followed by Tukey’s multiple comparisons test. Statistical significance was defined as *p*  < 0.05.

## 3. Results

### 3.1. Acquisition of 2372 DEGs‐KC From Monocytes, Macrophages, and CD4^+^ T Cells

After quality control, 23,579 cells and 18,223 genes were retained from the GSE151263 dataset. The top 2000 highly variable genes, such as C1QB, IGKC, and PPBP, were selected for downstream analyses. Fifteen PCs were selected for dimensionality reduction. Subsequent clustering grouped the cells into 17 distinct clusters, which were annotated into 14 cell types based on marker gene expression, including monocytes, naive T cells, and CD16^+^ monocytes (Figure 1A). The marker genes displayed specific high expression within each cluster (Figure 1B), confirming the accuracy of the cell type annotations. Among these clusters, CD16^+^ monocytes and naive T cells showed the highest proportions in ARDS samples, with macrophages exhibiting the greatest difference in abundance between the ARDS and control samples (Figure 1C). Given that monocytes can differentiate into macrophages, macrophages are key players in ARDS pathogenesis and the regulation of inflammatory responses [42, 43], and CD4^+^ T cells are critical regulators of immune function, these three cell types were selected as key cells for further analysis. Pathway enrichment analysis revealed that macrophages and monocytes shared many significantly enriched pathways. Specifically, macrophages were significantly enriched in pathways related to the transfer of lipopolysaccharide (LPS) from LPS‐binding protein (LBP) carriers to CD14^+^ cells, while monocytes were significantly enriched in the interleukin‐33 signaling pathway. CD4^+^ T cells were primarily enriched in pathways involving the 12‐hydroxylation of sterols by CYP8B1 (Figure 1D). These results highlight both the synergistic and distinct roles of these key cells in ARDS. Notably, the number and strength of intercellular communication were increased in ARDS samples compared to those in controls. In particular, the communication between CD4^+^ T cells and CD8^+^ T cells, CD16^+^ monocytes, B cells, and other immune cell types was significantly enhanced (Figure S1A,B). In both ARDS and control samples, the MIF–(CD74 + CXCR4) receptor–ligand pair played a major role in mediating interactions between key cells and other immune cells, such as CD4^+^ T cells with B cells and macrophages and macrophages with B cells and dendritic cells. In control samples, MIF (CD74 + CXCR4) was also a major mediator of interactions between monocytes and B cells or dendritic cells. In contrast, in ARDS samples, the RETN‐CAP1 receptor ligand pair was strongly involved in interactions between monocytes and CD16^+^ monocytes (Figure S2A–C). These results shed light on the enhanced intercellular communication and signaling mechanisms of key immune cells in ARDS.

Figure 1Analysis of single‐cell RNA‐seq data. (A) A uniform manifold approximation and projection (UMAP) plot showing 17 clusters annotated into 14 cell types. (B) Expression of marker genes across different cell clusters. (C) Proportions of various cell clusters in the ARDS and control groups. (D) Functional enrichment analysis of key cell types.

**Figure:**
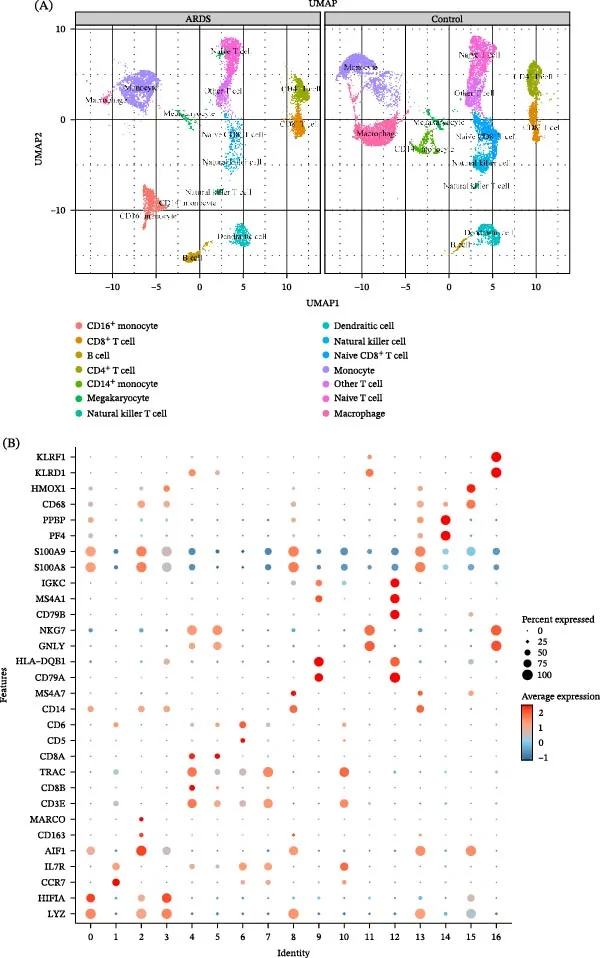

**Figure:**
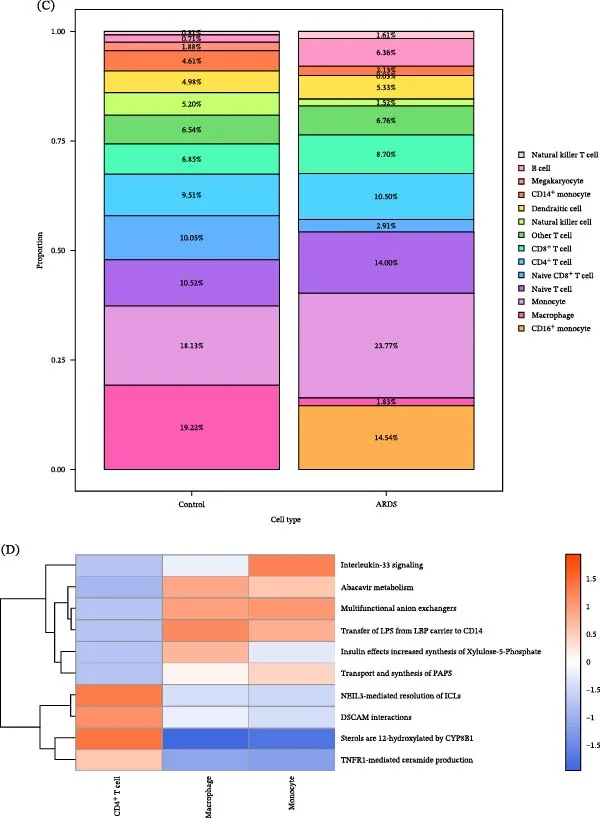

### 3.2. Candidate Genes Involved in ISR‐Related Pathways

**Figure 2:**
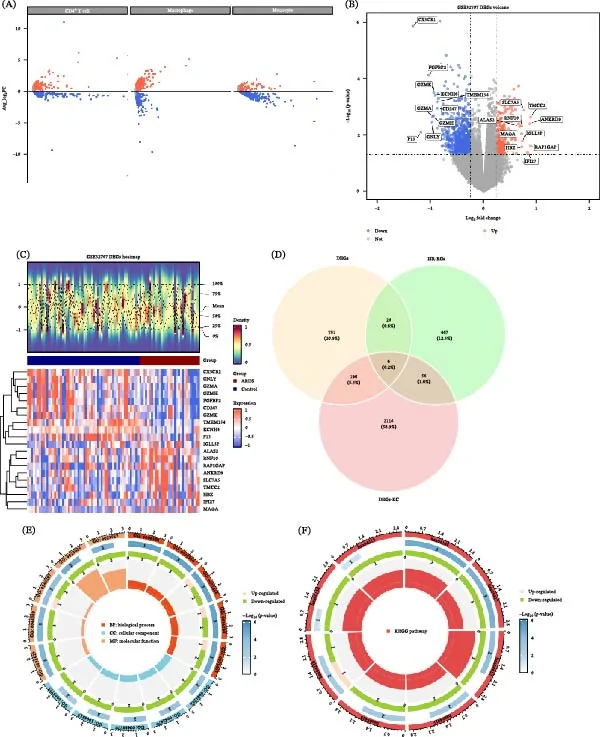
Differential gene expression analysis. (A) Volcano plot of differential gene expression for three cell types in the ARDS and control groups. (B) Volcano plot of overall differential gene expression between the ARDS and control groups. (C) Heatmap of differential gene expression between the ARDS and control groups. (D) Venn diagram of candidate genes. (E) Circular diagram of GO enrichment results for candidate genes. (F) Circular diagram of KEGG pathway enrichment results for candidate genes.

A total of 2372 DEGs‐KC were identified in the key cells, including 1087 upregulated and 1285 downregulated genes (Figure 2A). Additionally, 973 DEGs were detected between ARDS and control samples in the GSE32707 dataset, of which 236 genes were upregulated and 737 were downregulated in ARDS samples. The expression patterns of the top 20 DEGs exhibited obvious differences between the groups (Figure 2B,C). By intersecting the 529 ISR‐RGs, 2372 DEGs‐KC, and 973 DEGs, six candidate genes were identified (Figure 2D). These candidate genes were markedly enriched in 513 GO terms (*p* < 0.05), including 440 BPs, 41 CCs, and 32 MFs. Notably, they were mainly enriched in cellular response to hypoxia (BP), lysosomal lumen (CC), and MHC class II protein complex binding (MF) (Figure 2E). In addition, eight KEGG pathways were significantly enriched (*p* < 0.05), including protein processing in endoplasmic reticulum, lipid and atherosclerosis, and antigen processing and presentation pathways (Figure 2F). These results suggest that the candidate genes are closely related to ISR processes and immune responses in ARDS.

### 3.3. Nomogram Demonstrates Favorable Performance for ARDS Risk Stratification

The LASSO regression analysis showed that when the model reached the minimum error rate (lambda.min = 0.005328), five LASSO‐selected feature genes were identified, namely, HIPK2, HSPA8, NFE2L2, YY1, and ZFP36L1 (Figure 3A). Meanwhile, the importance and ranking of the candidate genes were also determined using the SVM‐RFE algorithm. When six genes were included, the model achieved its highest accuracy (YY1, HIPK2, NFE2L2, ZFP36L1, HSP90AA1, and HSPA8) (Figure 3B). The intersection of the two algorithms resulted in five shared feature genes: HIPK2, HSPA8, NFE2L2, YY1, and ZFP36L1 (Figure 3C). Among these, NFE2L2 and ZFP36L1 were significantly downregulated in ARDS samples in both GSE32707 and GSE66890 datasets and were therefore considered candidate ISR‐associated biomarkers for ARDS (Figure 3D,E). A nomogram was then constructed to predict the incidence of ARDS, showing that higher total scores corresponded to a higher probability of ARDS occurrence (Figure 3F). The calibration curve demonstrated good agreement between the predicted and actual values (Hosmer–Lemeshow test, *p* = 0.306), indicating strong predictive accuracy (Figure 3G). Furthermore, the CIC analysis showed that as the threshold probability increased (above 0.4), the model’s predictions gradually aligned with actual incidence rates, indicating robust clinical predictive performance (Figure 3H). Overall, the nomogram based on these biomarkers demonstrated favorable predictive performance and may provide an exploratory tool for ARDS risk stratification.

Figure 3Identification of hub genes using machine learning. (A) LASSO logistic regression: the coefficient plot (A1) and the λ selection plot (A2). (B) SVM‐RFE algorithm: the blue line indicates accuracy at different feature numbers; the *x*‐axis represents the number of genes, and the *y*‐axis indicates corresponding accuracy. (C) Venn diagram of candidate biomarkers. (D, E) Expression analysis of candidate biomarkers. (F) Nomogram model: points indicate individual scores for each variable; total points represent the sum of scores; and Pr indicates the probability of disease. (G) Calibration curve for nomogram model validation. (H) Clinical impact curve for nomogram model validation.  ^∗^ *p* < 0.05,  ^∗∗^ *p* < 0.01.

**Figure:**
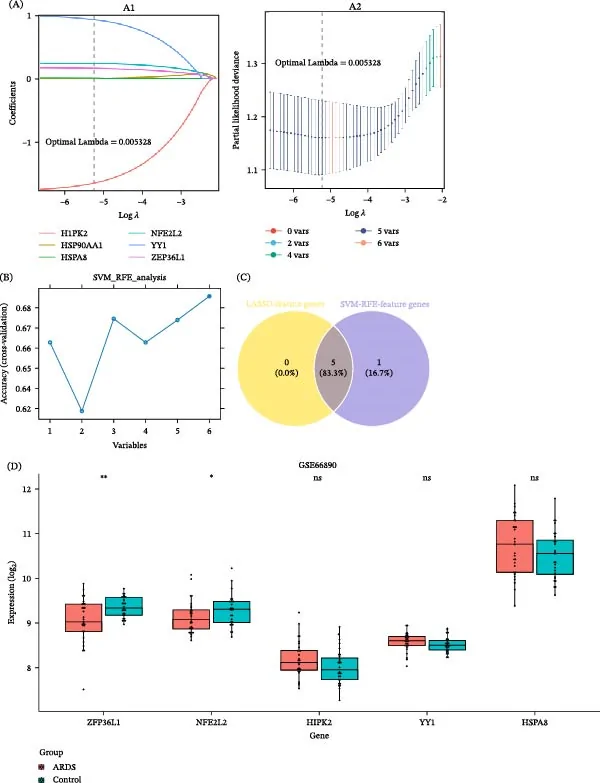

**Figure:**
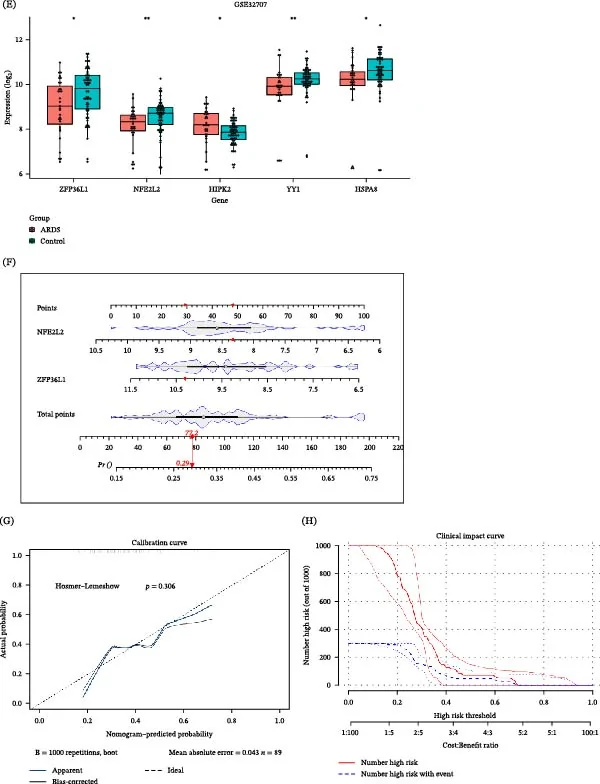

### 3.4. The Relationship Between Biomarkers and CLP‐Induced ARDS‐Like Lung Injury Validated by In Vivo Experiments

**Figure 4:**
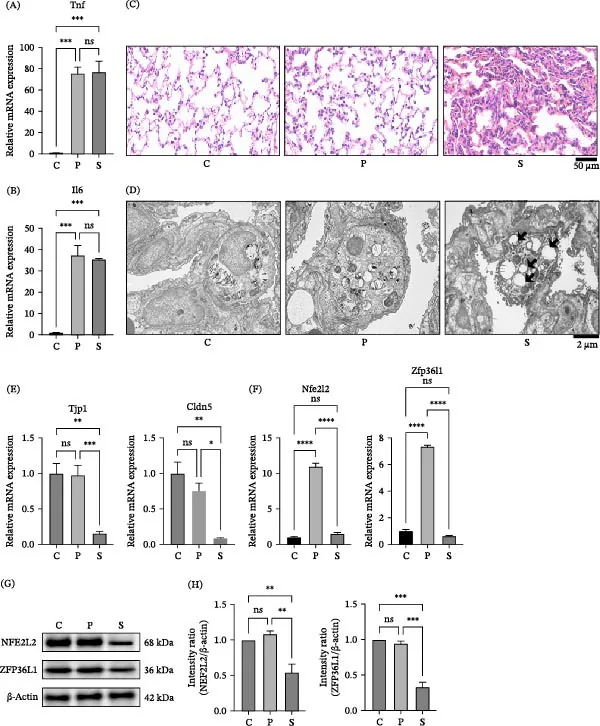
Experimental validation of inflammatory responses, lung injury, barrier disruption, and biomarker expression in the CLP‐based rat model. (A, B) qPCR analysis of Tnf and Il6 mRNA expression in peripheral blood. (C) Hematoxylin and eosin staining of lung tissues. Scale bar: 50 μm. (D) Transmission electron microscopy images of lung tissues; arrows indicate representative ultrastructural abnormalities, including vacuolated lamellar body‐like structures. Scale bar: 2 μm. (E) qPCR analysis of Tjp1 and Cldn5 mRNA expression in lung tissues. (F) qPCR analysis of Nfe2l2 and Zfp36l1 mRNA expression in lung tissues. (G, H) Representative western blot images and quantification of NFE2L2 and ZFP36L1 protein expression in lung tissues. Data are presented as mean ± SEM. Although four animals were included in each experimental group, three biological replicates per group were randomly selected for qPCR analyses (A, B, E, F) and western blot quantification (H). Statistical comparisons were performed using one‐way ANOVA followed by Tukey’s multiple comparisons test. ns, not significant;  ^∗^ *p* < 0.05,  ^∗∗^ *p* < 0.01,  ^∗∗∗^ *p* < 0.001,  ^∗∗∗∗^ *p* < 0.0001. C, control; P, cecal ligation and puncture group; S, cecal ligation and puncture + ARDS‐like lung injury group.

**Figure 5:**
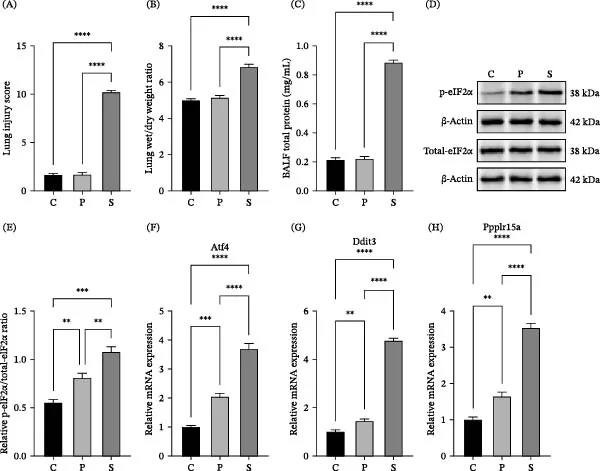
Quantitative assessment of lung injury and canonical ISR activation in the CLP‐induced ARDS‐like lung injury model. (A) Histological lung injury score based on hematoxylin and eosin‐stained lung sections. (B) Lung wet/dry weight ratio was measured to assess pulmonary edema. (C) BALF total protein concentration was measured as an indicator of alveolar–capillary barrier leakage. (D) Representative western blot images showing p‐eIF2α, total eIF2α, and their corresponding β‐actin loading controls in lung tissues. (E) Quantification of the relative p‐eIF2α/total eIF2α ratio after normalization to the corresponding β‐actin controls. (F–H) qPCR analysis of Atf4, Ddit3/Chop, and Ppp1r15 a/Gadd34 mRNA expression in lung tissues. p‐eIF2α and total eIF2α were detected on parallel membranes loaded with the same protein samples, and β‐actin was used as the loading control for each membrane. Data are presented as mean ± SEM. For (A–C) and (F–H), *n* = 4 biological replicates per group; for western blot quantification in (E), *n* = 3 biological replicates per group. Statistical comparisons were performed using one‐way ANOVA followed by Tukey’s multiple comparisons test.  ^∗∗^ *p* < 0.01,  ^∗∗∗^ *p* < 0.001,  ^∗∗∗∗^ *p* < 0.0001.

Compared with group C, peripheral blood Tnf and Il6 mRNA expression levels were significantly increased in groups P and S (*p* < 0.05) (Figure 4A,B). Optical microscopy and transmission electron microscopy revealed obvious lung tissue injury in group S, whereas no significant lung tissue damage was observed in groups C and P (Figure 4C,D). Additionally, Tjp1 and Cldn5 mRNA expression levels were significantly decreased in group S compared with groups C and P (*p* < 0.05) (Figure 4E). To further quantify pathological lung injury, pulmonary edema, and alveolar‐capillary barrier leakage, lung injury score, lung wet/dry weight ratio, and BALF total protein concentration were measured. The lung injury score was significantly increased in the S group compared with both the C and P groups (*p* < 0.05), whereas no significant difference was observed between the C and P groups (Figure 5A). Similarly, the S group showed a markedly increased wet/dry weight ratio compared with both the C and P groups (*p* < 0.05), with no significant increase in the P group compared with the C group (Figure 5B). BALF total protein concentration was also substantially elevated in the S group compared with both the C and P groups (*p* < 0.05), whereas no significant difference was observed between the C and P groups (Figure 5C). These findings provide quantitative evidence of histological lung injury, pulmonary edema, and increased alveolar‐capillary permeability in the CLP‐induced ARDS‐like lung injury group. Western blot analysis showed that the relative p‐eIF2α/total eIF2α ratio was significantly increased in the P group compared with the C group and was further elevated in the S group compared with the P group (*p*  < 0.05) (Figure 5D,E). To further assess canonical ISR activation in the CLP‐induced ARDS‐like lung injury model, the mRNA expression levels of Atf4, Ddit3/Chop, and Ppp1r15a/Gadd34 were examined by qPCR. Atf4 expression was significantly increased in the P group compared with the C group and was further elevated in the S group compared with the P group (*p*  < 0.05) (Figure 5F). Similarly, Ddit3/Chop and Ppp1r15a/Gadd34 mRNA levels were significantly upregulated in the P group compared with the C group and showed a further increase in the S group compared with the P group (*p*  < 0.05) (Figure 5G,H). These results indicate progressive activation of ISR‐related transcriptional responses during the transition from early CLP‐induced sepsis to established ARDS‐like lung injury. After confirming lung injury and ISR activation, we further examined the expression of the identified biomarkers. Notably, Nfe2l2 and Zfp36l1 mRNA expression levels were significantly higher in group P compared with groups C and S (*p*  < 0.05) (Figure 4F). Western blot analysis showed that NFE2L2 and ZFP36L1 protein levels were generally reduced in group S compared with those in group P (Figure 4G,H).

### 3.5. Alterations in Key Pathways and the Immune Microenvironment in ARDS

GSVA revealed that 14 KEGG pathways were significantly enriched. Among these, five pathways, including phenylalanine metabolism, glycine, serine, and threonine metabolism, lysine degradation, tyrosine metabolism, and porphyrin and chlorophyll metabolism, were activated in ARDS samples. In contrast, pathways, such as cell adhesion molecules (CAMs), natural killer cell‐mediated cytotoxicity, and T cell receptor signaling pathway, were inhibited in ARDS (Figure 6A). Subsequently, GSEA indicated that the biomarkers were commonly enriched in pathways, such as the spliceosome, oxidative phosphorylation, and lysosome, suggesting that NFE2L2 and ZFP36L1 may have coordinated roles in ARDS pathogenesis (Figure 6B,C).

Figure 6Pathway and immune infiltration analyses. (A) Gene set variation analysis (GSVA): the *x*‐axis shows *t* scores and the *y*‐axis lists pathways; red indicates pathways enriched in the ARDS group and blue indicates pathways enriched in the control group. (B, C) Gene set enrichment analysis (GSEA) results for NFE2L2 (B) and ZFP36L1 (C). (D) ssGSEA scoring heatmap: top panel shows groups and lower panel shows ssGSEA scores of immune cells across GSE32707 samples. Rows represent immune cells and columns represent samples. (E) Box plot of ssGSEA score difference for eight immune cell types between the ARDS and control groups. (F) Heatmap showing correlations among the eight immune cell types with differential ssGSEA scores. (G) Heatmap showing correlations between the two biomarkers and the eight immune cell types with differential ssGSEA scores.

**Figure:**
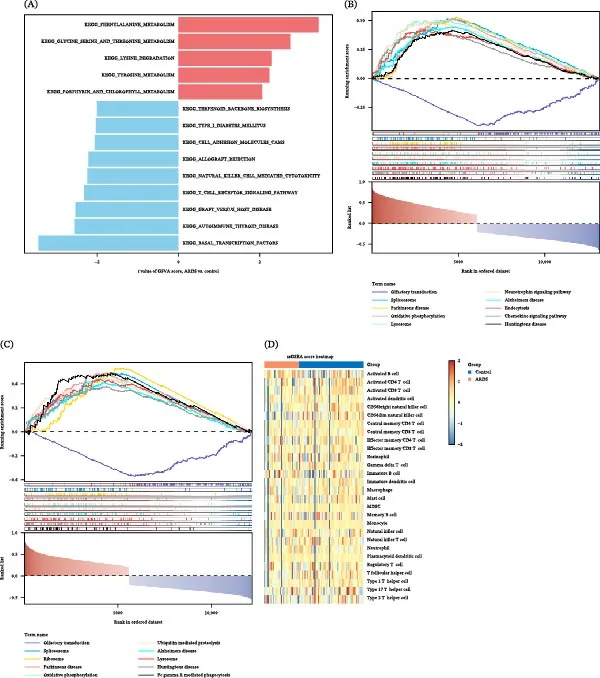

**Figure:**
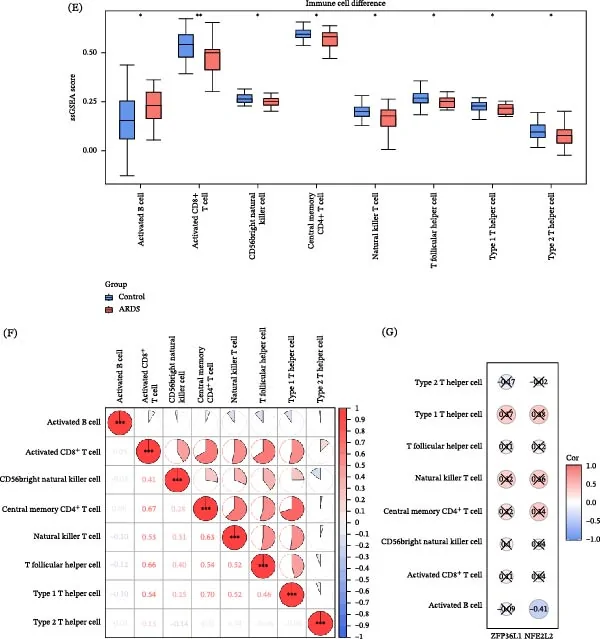

Moreover, the immune infiltration scores of different immune cells in ARDS and control samples are shown in Figure 6D. Eight immune cell types exhibited significant differences between ARDS and control samples (*p*  < 0.05), with only activated B cells showing higher immune scores in ARDS samples (Figure 6E). Interestingly, type 2 T helper cells showed weak correlations with other differential immune cells, and no strong negative correlations were observed among the remaining differential immune cell types. For instance, the strongest positive correlation was between type 1 T helper cells and central memory CD4^+^ T cells (correlation coefficient = 0.70, *p*  < 0.01) (Figure 6F). Notably, there was a significant negative correlation between activated B cells (high immune scores in ARDS) and NFE2L2 (correlation coefficient = −0.41, *p*  < 0.05) (Figure 6G). This suggests that NFE2L2 may modulate the immune microenvironment in ARDS by influencing activated B cells, thereby contributing to ARDS progression.

### 3.6. Localization Results for Biomarkers

**Figure 7:**
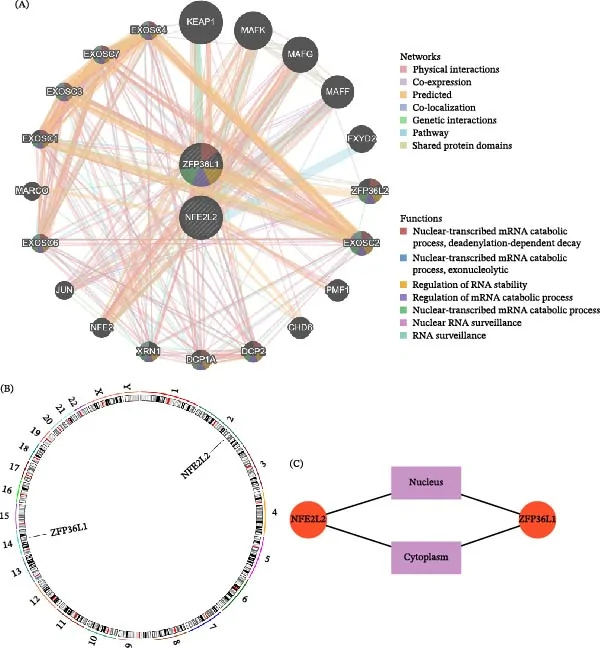
Functional association and regulatory network analyses. (A) GeneMANIA network showing functional interactions of biomarkers. (B) Chromosomal locations of biomarkers. (C) Subcellular localization of biomarkers.

Furthermore, the GGI analysis demonstrated that the interactions among NFE2L2, ZFP36L1, and 20 related genes were primarily based on physical interactions, co‐expression and predictive associations. ZFP36L1 showed functional similarity to ZFP36L2, DCP1A, and DCP2 and was involved in pathways such as the nuclear‐transcribed mRNA catabolic process, deadenylation‐dependent decay, regulation of RNA stability, and regulation of mRNA catabolic processes (Figure 7A). These insights help clarify how the biomarkers may function and offer potential avenues for targeting these pathways to treat ARDS. Additionally, ZFP36L1 and NFE2L2 were mapped to chromosomes 14 and 2, respectively (Figure 7B), providing important clues for understanding the genetic basis and molecular mechanisms underlying ARDS. Subcellular localization analysis further revealed that both ZFP36L1 and NFE2L2 were localized in the nucleus and cytoplasm (Figure 7C), consistent with their potential roles in transcriptional regulation and post‐transcriptional or stress‐response processes.

### 3.7. Uncovering the Complex Regulatory Network of ZFP36L1 and NFE2L2

**Figure 8:**
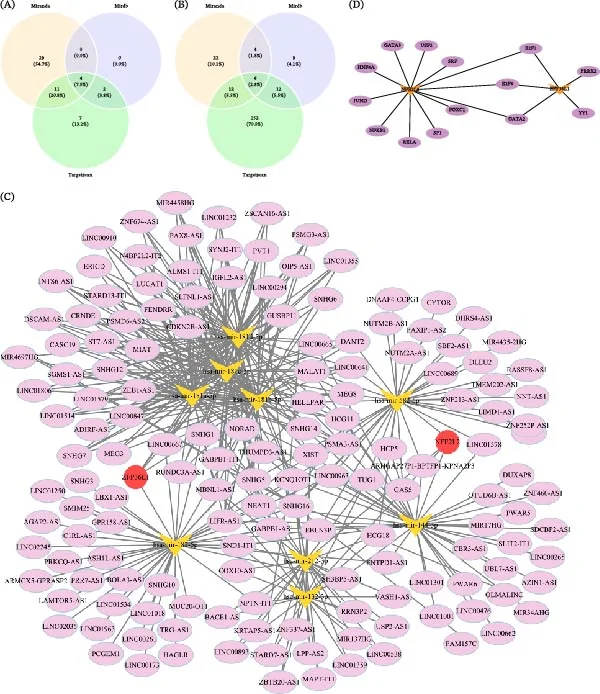
(A, B) Venn diagram of predicted miRNAs targeting biomarkers. (C) lncRNA‐miRNA‐mRNA network: red circles represent biomarkers, yellow arrows represent miRNAs, and pink ovals represent lncRNAs. (D) TF‐biomarker regulatory network.

By intersecting prediction results, four miRNAs targeting NFE2L2 and six miRNAs targeting ZFP36L1 were identified (Figure 8A,B). In addition, 152 upstream lncRNAs were found for these miRNAs, except for hsa‐miR‐4318, which did not have an identified upstream lncRNA. The resulting regulatory network consisted of 163 nodes and 430 interaction pairs. Although no miRNAs were found to co‐target both biomarkers, numerous shared lncRNAs targeting these miRNAs were identified, including DANT2, LINC00641, and MEG8 (Figure 8C). Furthermore, 12 TFs targeting NFE2L2 and five targeting ZFP36L1 were identified, with E2F1, E2F6, and GATA2 acting as common TFs, suggesting a potential for co‐regulation of the two biomarkers (Figure 8D). Overall, this regulatory network provides a foundation for future studies exploring the targeted modulation of biomarker expression in ARDS.

### 3.8. Pseudotime Expression of Key Cells and Biomarkers

To further investigate the mechanisms associated with key cells and biomarkers in ARDS at the single‐cell level, the expression patterns of ZFP36L1 and NFE2L2 were analyzed in detail. Both biomarkers were expressed across all cell types, with ZFP36L1 showing a relatively higher expression in monocytes and macrophages (Figure 9A). ZFP36L1 was significantly differentially expressed in key cells from both ARDS and control samples, whereas NFE2L2 displayed significant differential expression only in monocytes (*p*  < 0.0001) (Figure 9B,C). The expression dynamics of these biomarkers within key cells were then explored in more detail. Re‐annotation of CD4^+^ T cells revealed seven subclusters and five differentiation stages. CD4^+^ T‐cell predominantly occupied stage 1 during the early stages of differentiation, particularly in ARDS samples, while cells from control samples were mainly differentiated at later stages, primarily stages 4 and 5 (Figure 9D). Notably, pseudotime analysis showed that both NFE2L2 and ZFP36L1 had high expression levels during the early stages of CD4^+^ T‐cell differentiation, with ZFP36L1 displaying especially high expression in the early stages and a marked decrease in more differentiated cells (Figure 9E). In macrophages, 10 subclusters were identified, forming a single‐stage differentiation trajectory that displayed a “U”‐shaped pattern. In control samples, macrophages remained differentiated throughout the entire stage, whereas in ARDS samples, differentiation occurred mainly during the later stages (Figure 9F). The expression of NFE2L2 and ZFP36L1 remained high throughout macrophage differentiation, with both showing upregulation during the mid‐stage followed by downregulation in the late stage (Figure 9G). For monocytes, nine cellular subclusters spanning seven stages were identified. The early stages of monocyte differentiation were similar between ARDS and control samples; however, in the mid‐to‐late stages, control samples primarily differentiated into subclusters 3 and 4 (Figure 9H). Both NFE2L2 and ZFP36L1 maintained high expression levels throughout monocyte differentiation. Interestingly, their expression trends were similar during early differentiation but diverged in later stages. Specifically, NFE2L2 expression was significantly upregulated, while ZFP36L1 expression gradually decreased (Figure 9I). In summary, pseudotime modeling of key cells reveals their developmental trajectories and dynamic biomarker expression, providing a basis for future studies on stage‐specific biomarker dynamics in ARDS.

Figure 9Pseudotime trajectory analysis. (A) UMAP cluster map showing biomarker expression distribution. (B, C) Expression difference of biomarkers between the ARDS and control groups. (D) Pseudotime trajectory of CD4^+^ T cells. (E) Changes in biomarker expression in CD4^+^ T cells during differentiation. (F) Pseudotime trajectory of macrophages. (G) Changes in biomarker expression in macrophages during differentiation. (H) Pseudotime trajectory of monocytes. (I) Changes in biomarker expression in monocytes during differentiation.

**Figure:**
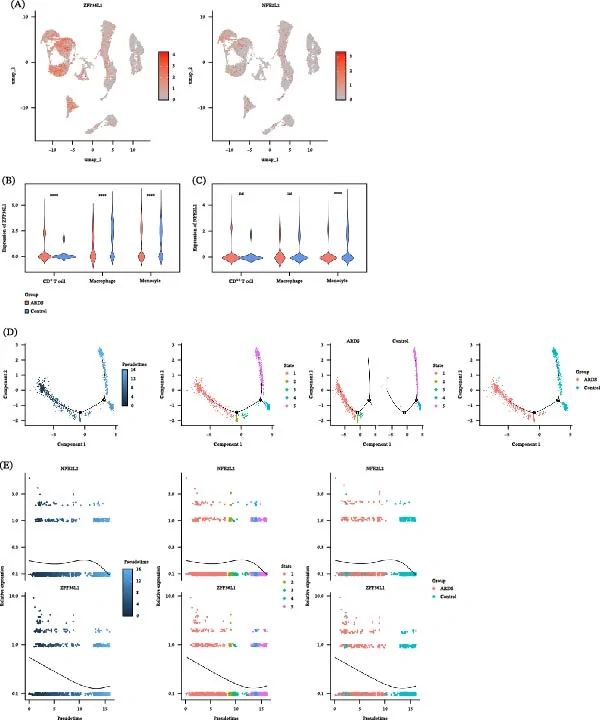

**Figure:**
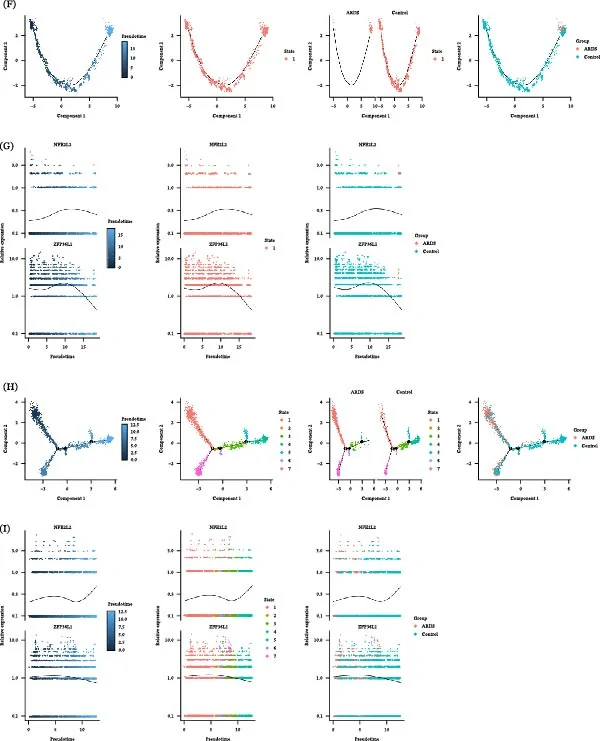

## 4. Discussion

ARDS is one of the most common and severe complications, characterized by complex pathophysiological mechanisms and high mortality, and currently lacks effective targeted therapies [44]. In recent years, increasing evidence has suggested a link between ARDS‐induced pathological changes and the ISR [14, 45]. As a cellular self‐protective mechanism activated under stress conditions, ISR modulates key pathways such as protein translation, oxidative stress, and immune responses, thereby participating in the development and progression of ARDS [46]. However, there is still a lack of ARDS‐specific biomarkers related to ISR, which limits its clinical application. In this study, by integrating transcriptomic data from the GEO database with scRNA‐seq data and validating findings through machine learning models and animal experiments, we identified two candidate ISR‐associated biomarkers for ARDS: NFE2L2 and ZFP36L1. The role of ISR in ARDS is likely stage‐dependent. During the early phase of sepsis‐induced lung injury, moderate ISR activation may help cells adapt to inflammatory, oxidative, and metabolic stress by restoring proteostasis and promoting stress tolerance [12, 13]. In contrast, sustained ISR activation during established ARDS‐like lung injury may reflect persistent cellular stress and may contribute to epithelial and endothelial barrier dysfunction, inflammatory amplification, and impaired repair [14–16]. In our CLP‐based model, canonical ISR activation markers, including the p‐eIF2α/total eIF2α ratio and Atf4, Ddit3/Chop, and Ppp1r15a/Gadd34 expression, progressively increased from early sepsis to ARDS‐like lung injury. These findings support the concept that ISR dynamics may be associated with disease progression and may provide a biologically relevant framework for biomarker‐based risk stratification and future therapeutic exploration. Importantly, NFE2L2 and ZFP36L1 should not be interpreted as canonical ISR markers themselves, but rather as candidate ISR‐associated biomarkers that may reflect stress‐response and immune‐regulatory programs occurring alongside canonical ISR activation, rather than direct readouts of ISR activity. Thus, the added canonical ISR markers support the presence of progressive ISR activation in the CLP‐based model, whereas the stage‐dependent changes in NFE2L2 and ZFP36L1 suggest that these genes may serve as candidate biomarkers of stress‐response and immune‐regulatory dysregulation accompanying ARDS progression.

NFE2L2, also known as NRF2, is a TF that plays a pivotal role in cellular antioxidant defense. When intracellular reactive oxygen species (ROS) levels increase, NFE2L2 translocates to the nucleus and binds to antioxidant response elements (AREs), thereby inducing the expression of downstream protective genes [47]. Previous studies have demonstrated that NFE2L2 (also known as NRF2) plays a critical role in the pathogenesis of ARDS. Genetic polymorphisms of NRF2, such as rs6721961, have been significantly associated with an increased risk of ARDS in patients with sepsis (OR = 1.93) [47]. Functional studies have shown that NRF2 deficiency heightens the sensitivity of alveolar epithelial cells to oxidative damage, exacerbates inflammatory cell infiltration, and increases pulmonary vascular permeability [48]. Protective mechanism research has revealed that ATF3 mitigates alveolar injury by stabilizing NRF2 through the inhibition of KEAP1‐mediated degradation [49]. Additionally, the mitochondria‐targeted antioxidant MitoQ has been shown to preserve endothelial barrier function and reduce ROS production via an NRF2‐dependent mechanism [50]. Thus, regulating NFE2L2 expression is emerging as a promising therapeutic strategy for lung injury and ARDS. These findings align with our results and support the potential involvement of NFE2L2 in stress‐response and immune‐regulatory mechanisms associated with ARDS.

ZFP36L1, also known as TIS11B, is a member of the ZFP36 zinc finger protein family and functions as an early response gene. To date, ZFP36L1 has not been directly reported in ARDS. However, evidence suggests that it promotes monocyte and macrophage differentiation by inhibiting CDK6 expression. Members of the ZFP36 family, including ZFP36L1, are known to cooperate in destabilizing cytokine mRNAs in activated CD4^+^ T cells, highlighting their role in modulating inflammatory responses. Notably, deficiency of ZFP36 family proteins has been shown to upregulate CREBBP, exacerbating ischemia‐reperfusion (I/R)‐induced lung injury, apoptosis, and inflammation and promoting I/R‐induced pulmonary fibrosis [51–53]. ZFP36L1 regulates inflammatory responses by modulating the stability of cytokine mRNAs and influencing monocyte/macrophage differentiation [51, 54]. These findings suggest that ZFP36L1 may help protect lung tissues from I/R injury, which is a common contributor to the development of ARDS.

The results of animal experiments showed that peripheral blood Tnf and Il6 mRNA expression levels were significantly increased in groups P and S compared with group C (*p*  < 0.05), confirming the presence of sepsis in these groups. Optical and electron microscopy revealed significant lung tissue injury in group S (*p* < 0.05), whereas no obvious lung tissue injury was observed in groups C and P. Additionally, Tjp1 and Cldn5 mRNA expression levels were reduced in group S, further indicating impairment of tight junction‐related barrier integrity in group S. Notably, Nfe2l2 and Zfp36l1 mRNA expression levels were significantly elevated in group P compared with groups C and S (*p*  < 0.05). These findings indicate that although the CLP 8‐h samples in group P did not develop ARDS‐like lung injury, they showed significantly increased expression of Nfe2l2 and Zfp36l1. In contrast, their mRNA expression decreased in group S, which developed ARDS‐like lung injury. Although the changes in Nfe2l2 and Zfp36l1 mRNA levels were not completely mirrored by their protein levels across all group comparisons, the western blot results generally supported reduced NFE2L2 and ZFP36L1 protein expression in the ARDS‐like stage compared with the early CLP stage. This partial discrepancy may reflect post‐transcriptional regulation, altered translational efficiency, protein stability, or time‐dependent regulation during the progression from early sepsis to established ARDS‐like lung injury. Together, these results support an association between NFE2L2/ZFP36L1 dysregulation and ARDS‐like lung injury.

In GSVA, five pathways were found to be activated in ARDS patients, with phenylalanine metabolism showing a strong association with ARDS. Previous studies have demonstrated that elevated phenylalanine levels promote pyroptosis of alveolar macrophages, which exacerbates lung inflammation and ARDS‐related mortality in mice. Similarly, metabolomic analyses of ARDS patients have shown that high phenylalanine levels may contribute to greater lung injury and higher mortality [55, 56]. In GSEA, the identified biomarkers were enriched in additional common pathways, such as the spliceosome, oxidative phosphorylation, and lysosome pathways, suggesting potential coordinated roles of NFE2L2 and ZFP36L1 in ARDS. Notably, mitochondrial dysfunction in ARDS can elevate intracellular oxidative stress and inflammation, thus aggravating lung injury. These findings suggest that NFE2L2 and ZFP36L1 may participate in stress‐response and immune‐regulatory processes occurring in the context of ISR activation in ARDS [57]. Although direct immunofluorescence‐based co‐localization experiments were not performed in the present study, the subcellular localization analysis showed that both NFE2L2 and ZFP36L1 were localized in the nucleus and cytoplasm. In addition, regulatory network analysis identified shared upstream regulatory elements, including common TFs, such as E2F1, E2F6, and GATA2, suggesting a potential basis for coordinated regulation. Together with their partially overlapping pathway enrichment and dynamic expression patterns in key immune cell populations, these findings suggest that NFE2L2 and ZFP36L1 may act in a coordinated manner during ARDS progression. However, whether these two biomarkers directly interact, co‐localize at the protein level, or regulate each other requires further experimental validation.

Analysis of immune infiltration revealed eight differential immune cell types in ARDS and control samples (*p* < 0.05), with activated B cells uniquely showing higher infiltration in ARDS. It has been reported that in sepsis‐induced ARDS, the peripheral blood immune cell composition, including B cells, undergoes significant changes as the disease progresses. In a murine model of LPS‐induced ALI, B‐cell‐specific loss of IL‐10 resulted in increased proinflammatory cytokine production, persistent leukocyte infiltration, and prolonged disruption of the alveolar barrier. These findings highlight the critical role of B‐cell‐derived IL‐10 in resolving LPS‐induced ALI and suggest that it may serve as a potential therapeutic target [58, 59]. In addition, our study found a significant negative correlation between NFE2L2 expression and activated B‐cell infiltration (correlation coefficient = −0.41), suggesting that NFE2L2 may influence ARDS progression by modulating activated B‐cell levels.

Furthermore, the number and strength of cell–cell interactions were increased in ARDS samples, particularly involving key immune cells. Notably, macrophages showed enhanced communication with B cells, and monocytes exhibited increased interactions with both CD4^+^ T cells and B cells. In addition, CD4^+^ T cells demonstrated greater crosstalk with multiple cell types, especially B cells, while showing reduced interaction with monocytes. Prior studies have shown that B cells play an important role in regulating immune responses in ARDS through the production of IL‐10, a key anti‐inflammatory cytokine. Moreover, increased B cell numbers have been associated with improved survival in ARDS patients, likely due to their regulatory effects on immune regulation and inflammatory response [58, 60]. Altogether, these alterations in intercellular communication appear to be closely related to ARDS pathogenesis and progression.

The mechanisms involving key cells and biomarkers in ARDS were further explored at the single‐cell level. ZFP36L1 and NFE2L2 were expressed across all cell types, with ZFP36L1 showing a relatively higher expression in monocytes and macrophages. ZFP36L1 was significantly differentially expressed in key cell populations in both ARDS and control samples, while NFE2L2 showed a marked differential expression specifically in monocytes. In macrophages, the differentiation trajectory displayed a characteristic “U” shape. Notably, macrophages in control samples differentiated steadily throughout, whereas those in ARDS samples differentiated predominantly at later stages. The expression of both NFE2L2 and ZFP36L1 remained high throughout macrophage differentiation, with an increase during intermediate stages, followed by downregulation at later stages. For monocytes, early differentiation stages were similar between ARDS and controls; however, NFE2L2 expression increased significantly in late stages, while ZFP36L1 expression declined. Pseudotime single‐cell analysis enabled the identification of key cell subpopulations, their state transitions, and the dynamic expression of these biomarkers during ARDS progression, providing insight into cellular heterogeneity and disease pathogenesis. These findings provide a temporal framework for future studies of stage‐specific biomarker dynamics in ARDS.

In this study, we identified two candidate ISR‐associated biomarkers in sepsis‐induced ARDS using multiple analytical approaches and experimental validation. From a translational perspective, peripheral blood NFE2L2 and ZFP36L1 expression may provide a candidate biomarker framework for ARDS risk stratification, dynamic disease monitoring, and future molecular stratification of sepsis‐induced ARDS, particularly when combined with clinical parameters in prospective cohorts. Functional pathways and potential molecular mechanisms were revealed through single‐gene GSEA, immune infiltration analysis, and regulatory network construction. Furthermore, single‐cell analysis indicated the expression dynamics of these biomarkers in three key cell types, providing a theoretical basis for future biomarker‐guided and mechanistic studies in sepsis‐induced ARDS.

Although this study supports the potential of NFE2L2 and ZFP36L1 as candidate biomarkers for ARDS through integrated bioinformatics analyses and animal experiments, several limitations remain. First, the study primarily relied on transcriptomic data from public databases, which were limited in sample size and clinical heterogeneity. Independent clinical PBMC samples from patients with sepsis‐induced ARDS were not collected for qPCR validation of NFE2L2 and ZFP36L1 expression, which should be addressed in future prospective clinical studies. In addition, detailed timing information for sample collection relative to sepsis diagnosis, ARDS onset, ICU admission, or mechanical ventilation initiation was not consistently available in these public datasets, which limited our ability to evaluate temporal ISR dynamics across different clinical stages. This issue should be addressed in future prospective studies with standardized sampling time points to clarify the temporal relationship between ISR activation, biomarker expression, and ARDS progression. Second, the animal experiments were based solely on the CLP‐induced ARDS‐like lung injury model without validation in other models. Third, the precise molecular mechanisms linking these two genes to ISR‐associated stress‐response and immune‐regulatory processes have yet to be fully elucidated. The lack of functional experiments, such as gene knockdown or overexpression of ZFP36L1, limits the definitive mechanistic interpretation in the present study. To address these limitations, future research should focus on several key areas. First, we plan to expand clinical validation through multicenter, prospective cohort studies that include ARDS patients with diverse etiologies, with standardized PBMC collection and processing protocols and qPCR validation of NFE2L2 and ZFP36L1 expression. Second, to investigate underlying mechanisms and potential coordinated regulatory interactions, we will generate conditional knockout rat models using gene‐editing technologies and employ immunofluorescence co‐localization, double‐label staining, gene perturbation approaches in macrophage/monocyte models, and single‐cell and spatial transcriptomics to systematically characterize the roles of NFE2L2 and ZFP36L1 in inflammatory cytokine regulation, monocyte/macrophage differentiation, and different pathological stages of ARDS. From a translational perspective, we aim to develop rapid detection platforms based on nanomaterials to evaluate the clinical utility of these biomarkers in early warning, disease monitoring, and prognosis assessment. In addition, we will explore candidate molecular strategies targeting these pathways to assess their translational potential. These efforts will not only enhance our understanding of ARDS pathogenesis but also provide novel biomarkers and therapeutic targets for clinical management.

## 5. Conclusion

This study identified NFE2L2 and ZFP36L1 as candidate ISR‐associated biomarkers in sepsis‐induced ARDS through integrated bioinformatics and experimental validation. Their roles in oxidative stress, inflammation, immune regulation, and cell differentiation were substantiated by GSEA, immune infiltration, cell–cell communication, and single‐cell trajectory analyses. Collectively, these findings provide a theoretical framework for biomarker‐driven risk stratification and future mechanistic studies in ARDS, highlighting the potential of NFE2L2 and ZFP36L1 as candidate biomarkers and exploratory therapeutic targets.

**Nomenclature**
ARDS: Acute respiratory distress syndrome
ISR: Integrated stress response
ISR‐RGs: Integrated stress response‐related genes
PBMC: Peripheral blood mononuclear cell
DEGs: Differentially expressed genes
scRNA‐seq: Single‐cell RNA sequencing
GO: Gene Ontology
KEGG: Kyoto Encyclopedia of Genes and Genomes
GSVA: Gene set variation analysis
GSEA: Gene set enrichment analysis
CLP: Cecal ligation and puncture.

## Author Contributions

Ye Gao conceived and designed the study, performed the bioinformatics analyses, and drafted the manuscript. Ni Wang conducted the animal experiments, including CLP/ARDS‐like lung injury modeling, sample collection, and molecular assays, and contributed to data acquisition and interpretation. Zhaoyang Xiao and Ni Wang supervised the project and provided critical revisions.

## Funding

This study was supported by the Beijing Life Oasis Public Welfare Service Center (Grant HXLX20240041).

## Disclosure

Zhaoyang Xiao and Ni Wang have approved the final version for submission. The funder had no role in the study design, data collection and analysis, decision to publish, or preparation of the manuscript. No cell lines were used in this study.

## Ethics Statement

All animal procedures were approved by the Laboratory Animal Ethics Committee of Dalian Medical University (Approval Number XL250911369) and were conducted in accordance with institutional guidelines and the NIH Guide for the Care and Use of Laboratory Animals. This study did not involve human participants; therefore, informed consent was not required.

## Consent

The authors have nothing to report.

## Conflicts of Interest

The authors declare no conflicts of interest.

## Supporting Information

Additional supporting information can be found online in the Supporting Information section.

## Supporting information


**Supporting Information** Table S1: List of 529 integrated stress response‐related genes compiled from previously published literature after removal of duplicates. Figure S1: Cell–cell communication networks in the control and ARDS groups. (A) Communication network among cells in the control group. (B) Communication network among cells in the ARDS group. Figure S2: Ligand–receptor signaling patterns received by key immune cells. (A) Signaling received by CD4^+^ T cells. (B) Signaling received by macrophages. (C) Signaling received by monocytes.

## Data Availability

Publicly available datasets were analyzed in this study. The microarray datasets GSE32707 and GSE66890 and the scRNA‐seq dataset GSE151263 are available in the Gene Expression Omnibus (GEO). Additional data are available from the corresponding author upon reasonable request.
