# Supplementary material for: Identification of Integrated Stress Response–Related Biomarkers in Sepsis‐Induced Acute Respiratory Distress Syndrome
Source: Mediators Inflamm. 2026 Sep 7;2026:3190064. doi: 10.1155/mi/3190064 (PMC13548138; doi:10.1155/mi/3190064)
Supplement: Supplementary file 1 [file MI-2026-3190064-s001.zip › 4.Supplementary_Material_Legends.docx]

**Supplementary Materials**

**Supplementary Figure S1. Cell-cell communication networks in the control and ARDS groups.**

(A) Communication network among cells in the control group. (B) Communication network among cells in the ARDS group.

**Supplementary Figure S2.** **Ligand-receptor signaling patterns received by key immune cells.**

(A) Signaling received by CD4+ T cells. (B) Signaling received by macrophages. (C) Signaling received by monocytes.

**Supplementary Table S1.**

List of 529 integrated stress response-related genes compiled from previously published literature after removal of duplicates.
